# Deficient nitric oxide signalling impairs skeletal muscle growth and performance: involvement of mitochondrial dysregulation

**DOI:** 10.1186/s13395-014-0022-6

**Published:** 2014-12-12

**Authors:** Clara De Palma, Federica Morisi, Sarah Pambianco, Emma Assi, Thierry Touvier, Stefania Russo, Cristiana Perrotta, Vanina Romanello, Silvia Carnio, Valentina Cappello, Paolo Pellegrino, Claudia Moscheni, Maria Teresa Bassi, Marco Sandri, Davide Cervia, Emilio Clementi

**Affiliations:** Unit of Clinical Pharmacology, National Research Council-Institute of Neuroscience, Department of Biomedical and Clinical Sciences “Luigi Sacco”, University Hospital “Luigi Sacco”, Università di Milano, Milano, Italy; Scientific Institute IRCCS Eugenio Medea, Bosisio Parini, Italy; Dulbecco Telethon Institute at Venetian Institute of Molecular Medicine, Padova, Italy; National Research Council-Institute of Neuroscience, Department of Medical Biotechnology and Translational Medicine, Università di Milano, Milano, Italy; CNI@NEST, Italian Institute of Technology, Pisa, Italy; Unit of Morphology, Department of Biomedical and Clinical Sciences “Luigi Sacco”, Università di Milano, Milano, Italy; Department of Biomedical Science, Università di Padova, Padova, Italy; Department for Innovation in Biological, Agro-food and Forest Systems, Università della Tuscia, Viterbo, Italy

**Keywords:** Nitric oxide synthase and signalling, Mitochondrial bioenergetics, Mitochondrial network, Unfolded protein response, Autophagy, Akt-mTOR pathway, Akt-FoxO3-Mul-1 axis, Fibre growth, Muscle structure, Muscle exercise

## Abstract

**Background:**

Nitric oxide (NO), generated in skeletal muscle mostly by the neuronal NO synthases (nNOSμ), has profound effects on both mitochondrial bioenergetics and muscle development and function. The importance of NO for muscle repair emerges from the observation that nNOS signalling is defective in many genetically diverse skeletal muscle diseases in which muscle repair is dysregulated. How the effects of NO/nNOSμ on mitochondria impact on muscle function, however, has not been investigated yet.

**Methods:**

In this study we have examined the relationship between the NO system, mitochondrial structure/activity and skeletal muscle phenotype/growth/functions using a mouse model in which nNOSμ is absent. Also, NO-induced effects and the NO pathway were dissected in myogenic precursor cells.

**Results:**

We show that nNOSμ deficiency in mouse skeletal muscle leads to altered mitochondrial bioenergetics and network remodelling, and increased mitochondrial unfolded protein response (UPR^mt^) and autophagy. The absence of nNOSμ is also accompanied by an altered mitochondrial homeostasis in myogenic precursor cells with a decrease in the number of myonuclei per fibre and impaired muscle development at early stages of perinatal growth. No alterations were observed, however, in the overall resting muscle structure, apart from a reduced specific muscle mass and cross sectional areas of the myofibres. Investigating the molecular mechanisms we found that nNOSμ deficiency was associated with an inhibition of the Akt-mammalian target of rapamycin pathway. Concomitantly, the Akt-FoxO3-mitochondrial E3 ubiquitin protein ligase 1 (Mul-1) axis was also dysregulated. In particular, inhibition of nNOS/NO/cyclic guanosine monophosphate (cGMP)/cGMP-dependent-protein kinases induced the transcriptional activity of FoxO3 and increased Mul-1 expression. nNOSμ deficiency was also accompanied by functional changes in muscle with reduced muscle force, decreased resistance to fatigue and increased degeneration/damage post-exercise.

**Conclusions:**

Our results indicate that nNOSμ/NO is required to regulate key homeostatic mechanisms in skeletal muscle, namely mitochondrial bioenergetics and network remodelling, UPR^mt^ and autophagy. These events are likely associated with nNOSμ-dependent impairments of muscle fibre growth resulting in a deficit of muscle performance.

**Electronic supplementary material:**

The online version of this article (doi:10.1186/s13395-014-0022-6) contains supplementary material, which is available to authorized users.

## Background

Nitric oxide (NO) is a gas and a messenger with pleiotropic functions in most tissues and organs, synthesized by a family of NO synthases. NO is also generated in skeletal muscle, in particular by the muscle-specific neuronal NO synthases (nNOS or NOS1) [1,2]. nNOSμ is the predominant nNOS isoform in muscle and is anchored to the sarcolemma as a component of the dystrophin glycoprotein complex [3]. This enzyme produces NO at low, physiological levels (in the pico to nanomolar range) in a way controlled by second messengers [1,2]; its expression is increased by crush injury, muscle activity and ageing [4,5]. NO has an important role in regulating skeletal muscle physiological activity, including excitation-contraction coupling, muscle force generation, auto-regulation of blood flow, calcium homeostasis, metabolism and bioenergetics [2,6,7]. In addition, it is a key determinant in myogenesis that it regulates at several key steps, especially when the process is stimulated to repair muscle damage after injury [5,8,9].

The importance of NO in muscle repair also emerges from the observation that nNOS signalling is defective in many genetically diverse skeletal muscle diseases in which muscle repair is dysregulated, including Duchenne muscular dystrophy, Becker muscular dystrophy, limb-girdle muscular dystrophies 2C, 2D and 2E, Ullrich congenital muscular dystrophy and inflammatory myositis [3,10-13]. Based on this evidence and on the fact that the restoration of NO signalling by nNOS overexpression ameliorates muscle function [14,15], genetic and pharmacologic strategies to boost nNOS/NO signalling in dystrophic muscle are being tested with encouraging results: in particular, the combination of NO donation with non steroidal anti-inflammatory activity limits muscle damage and favours muscle healing *in vivo* [16-18] such that it is currently being tested as a therapeutic for Duchenne muscular dystrophy in humans [19,20].

The observation that nNOS is localised in close proximity to mitochondria suggests a tight coupling between NO generation and regulation of mitochondrial respiration and metabolism. The role of NO in regulating oxidative phosphorylation and mitochondrial biogenesis in skeletal muscle physiology has been established [21-24]. Likewise NO-dependent inhibition of mitochondrial fission occurs during myogenic differentiation [25].

How the effects of NO on mitochondria impact on muscle function, however, has not been investigated yet. Elucidation of this aspect is relevant in view of the role that mitochondria play in muscle pathophysiology and may shed light on the muscular disorders in which NO signalling is impaired [26]. In particular, increases in mitochondria number and oxidative phosphorylation activity is relevant during differentiation [27] and the balance of fission and fusion is necessary to preserve excitation contraction coupling and prevent atrophy [28,29]. In addition, mitochondria are involved in regulating autophagy [30], whose derangement plays a role in a number of inherited muscle diseases [31-33]. Mitochondrial protein homeostasis is maintained through proper folding and assembly of polypeptides. This involves the mitochondrial unfolded protein response (UPR^mt^), a stress response that activates transcription of nuclear-encoded mitochondrial chaperone genes to maintain proteins in a folding or assembly-competent state, preventing deleterious protein aggregation [34-36].

In this study we have examined the relationship between the NO system, mitochondrial structure/activity and skeletal muscle phenotype/growth/functions using a mouse model in which nNOSμ is absent (NOS1-/-). Also, NO-induced effects and the NO pathway were dissected in myogenic precursor cells. Our results indicate that the deficit in NO signalling leads in skeletal muscle to alterations in mitochondrial morphology, bioenergetics and network remodelling, accompanied by defective autophagy and the induction of a UPR^mt^ response. These events, while not severely altering the overall resting skeletal muscle structure, are associated with modifications in the Akt-mammalian target of rapamycin (mTOR) pathway and Akt-FoxO3-mitochondrial E3 ubiquitin protein ligase 1 (Mul-1) axis and are sufficient to dysregulate skeletal muscle growth and exercise performance.

## Methods

### Animals

NOS1-/- animals are mice homozygous for targeted disruption of the nNOS gene (strain name B6129S4-NOS1^tm1Plh^/J) that were purchased from Jackson Laboratories (Bar Harbor, Maine, USA) (stock no. 002633). In this mouse line, targeted deletion of exon 2 specifically eliminates expression of nNOSμ [37]. NOS1-/- mice were crossed with the wild-type B6129 to maintain the original background and to obtain a colony of NOS1-/- mice and wild-type littermate controls, with genotyping performed from tail clippings. Experiments were performed on male mice at postnatal day 10 (P10) and P120. C57BL/6 wild-type mice (strain name C57Bl10SnJ) were purchased from Charles River (Calco, Italy). Animals were housed in a regulated environment (23 ± 1°C, 50 ± 5% humidity) with a 12-hour light/dark cycle (lights on at 08.00 a.m.), and provided with food and water *ad libitum*. For specific experiments, mice were killed by cervical dislocation. All studies were conducted in accordance with the Italian law on animal care N° 116/1992 and the European Communities Council Directive EEC/609/86. The experimental protocols were approved by the Ethics Committee of the University of Milano. All efforts were made to reduce both animal suffering and the number of animals used.

### Mitochondrial membrane potential

Mitochondrial membrane potential in isolated transfected fibres from *flexor digitorum brevis* muscles was measured by epifluorescence microscopy based on the accumulation of tetramethylrhodamine methyl ester (TMRM) fluorescence [25,29,38]. Briefly, *flexor digitorum brevis* myofibres were placed in 1 ml Tyrode’s buffer and loaded with 5 nM TMRM supplemented with 1 μM cyclosporine H for 30 minutes at 37°C. Myofibres were then observed with an Olympus IX81 inverted microscope equipped with a CellR imaging system (Olympus, Tokio, Japan). Sequential images of TMRM fluorescence were acquired every 60 seconds with a × 20 0.5, UPLANSL N A objective (Olympus). When indicated, oligomycin (5 μM) or the protonophore carbonylcyanide-p-trifluoromethoxyphenyl hydrazone (FCCP, 4 μM) was added [39]. Images were acquired and stored, and analysis of TMRM fluorescence over mitochondrial regions of interest was performed using ImageJ software (http://rsbweb.nih.gov/ij/).

### Primary myogenic cell cultures

Using published protocols [25], myogenic precursor cells (satellite cells) were freshly isolated from the muscles of newborn C57BL/6 mice. When indicated, cells were obtained from NOS1-/- mice and wild-type littermate controls. Briefly, hind limb muscles were digested with 2% collagenase-II and dispase for 10 minutes at 37°C with gentle agitation. Contamination by non-myogenic cells was reduced by pre-plating the collected cells onto plastic dishes where fibroblasts tend to adhere more rapidly. Dispersed cells were then resuspended in Iscove’s modified Dulbecco’s medium supplemented with 20% foetal bovine serum, 3% chick embryo extract (custom made), 10 ng/ml fibroblast growth factor, 100 U/ml penicillin, 100 μg/ml streptomycin and 50 μg/ml gentamycin, and plated onto matrigel-coated dishes. Differentiation was induced by changing the medium to Iscove’s modified Dulbecco’s medium supplemented with 2% horse serum and the antibiotics.

### Measurement of ATP formation

*Tibialis anterior* and diaphragm muscles were dissected, trimmed clean of visible fat and connective tissue, minced with scissors and digested in ATP medium, containing 50 mM Tris-HCl (pH 7.4), 100 mM KCl, 5 mM MgCl_2_, 1.8 mM ATP, 1 mM ethylenediaminetetraacetic acid (EDTA), and 0.1% collagenase type V for 10 minutes at 37°C under strong agitation. After centrifugation, the pellet was homogenised with Ultra-Turrax T10 (Ika-lab, Staufen, Germany) for 10 seconds at maximum speed in ATP medium. The mitochondrial fraction, obtained by different centrifugations (380 *g* and 10,000 *g* for five minutes at 4°C), was then suspended in a mitochondria resuspension buffer containing 12.5 mM Tris acetate, 225 mM sucrose, 44 mM KH_2_PO_4_ and 6 mM EDTA. Total oxidative phosphorylation (OXPHOS)-ATP in isolated mitochondria was measured by the luciferin-luciferase method, as described, with slight modifications [25]. Briefly, mitochondria were plated in 96 wells and treated with buffer-A (150 mM KCl, 25 mM Tris-HCl, 2 mM EDTA, 0.1% bovine serum albumin, 10 mM KH_2_PO_4_ and 0.1 mM MgCl_2_ (pH 7.4) containing 0.8 M malate, 2 M glutamate, 500 mM ADP, 100 mM luciferin and 1 mg/ml luciferase. Oligomycin (2 μg/ml) was also used to detect the presence of glycolytic ATP. OXPHOS-ATP was measured using a GloMax luminometer (Promega, Milan, Italy).

### High-resolution respirometry

Respiratory chain defects were assessed in *tibialis anterior* and diaphragm fibre bundles using published protocols [40-42]. After transferring the tissue sample into ice-cold BIOPS (10 mM CaK_2_ ethyleneglycoltetraacetic acid (EGTA) buffer, 7.23 mM K_2_ EGTA buffer, 0.1 μM free calcium, 20 mM imidazole, 20 mM taurine, 50 mM 2-(N-morpholino)ethanesulfonic acid hydrate, 0.5 mM dithiothreitol, 6.5 mM MgCl_2_ 6H_2_O, 5.7 mM ATP and 15 mM phosphocreatine (pH 7.1)), connective tissue was removed and the muscle fibres were mechanically separated. Complete permeabilisation of the plasma membrane was ensured by gentle agitation for 30 minutes at 4°C in 2 ml of BIOPS solution containing 50 μg/ml saponin. The fibre bundles were rinsed by agitation for 10 minutes in ice-cold mitochondrial respiration medium (MiR05; 0.5 mM EGTA, 3 mM MgCl_2_, 60 mM K-lactobionate, 20 mM taurine, 10 mM KH_2_PO_4_, 20 mM Hepes, 110 mM sucrose and 1 g/l bovine serum albumin (pH 7.1). The permeabilised muscle fibres were weighed and added to an Oxygraph-2 k respiratory chamber (Oroboros Instruments, Innsbruck, Austria) containing 2 ml of MiR06 (MiR05 supplemented with 280 U/ml catalase at 37°C). Oxygen flux per muscle mass was recorded online using DatLab software (Oroboros Instruments). After calibration of the oxygen sensors at air saturation, a few μl of H_2_O_2_ were injected into the chamber to reach a concentration of 400 μM O_2_. In order to detect the electron flow through CI and CII mitochondrial complexes, titrations of all of substrates, uncouplers and inhibitors were added in series as previously described [41,42]. The measurement of CIV respiration was obtained by addition of the artificial substrates N,N,N’,N’-tetramethyl-p-phenylenediamine dihydrochloride and ascorbate [40]. Oxygen fluxes were corrected by subtracting residual oxygen consumption from each measured mitochondrial steady-state. Respirometry measurements were performed in duplicate on each specimen.

### Real-time quantitative PCR

Satellite cells and muscle tissue samples were homogenised, and RNA was extracted using the TRIzol protocol (Invitrogen-Life Technologies, Monza, Italy). Using published protocols [43], after solubilisation in RNase-free water, first-strand cDNA was generated from 1 μg of total RNA using the ImProm-II Reverse Transcription System (Promega). As show in Table 1, a set of primer pairs amplifying fragments ranging from 85 to 247 bp was designed to hybridise to unique regions of the appropriate gene sequence. Real-time quantitative PCR (qPCR) was performed using the SYBR Green Supermix (Bio-Rad, Hercules, CA, USA) on a Roche LightCycler 480 Instrument (Roche, Basel, Switzerland). All reactions were run in triplicate. A melt-curve analysis was performed at the end of each experiment to verify that a single product per primer pair was amplified. As a control experiment, gel electrophoresis was performed to verify the specificity and size of the amplified qPCR products. Samples were analysed using the Roche LightCycler 480 software and the second derivative maximum method. The fold increase or decrease was determined relative to a calibrator after normalising to 36b4 (internal standard) through the use of the formula 2^-ΔΔCT^ [44].

**Table 1:** **Primer pairs designed for qPCR analysis**

**Name/symbol**	**Gene accession Number**	**Primer sequence**	**Amplicon**
*Atg4b*	NM_174874	F: 5′-ATTGCTGTGGGGTTTTTCTG-3′	247 bp
		R: 5′-AACCCCAGGATTTTCAGAGG-3′	
*Atrogin-1 (fbxo32)*	NM_026346	F: 5′-GCAAACACTGCCACATTCTCTC-3′	93 bp
		R: 5′-CTTGAGGGGAAAGTGAGACG-3′	
*Bnip3*	NM_009760	F: 5′-TTCCACTAGCACCTTCTGATGA-3′	150 bp
		R: 5′-GAACACGCATTTACAGAACAA-3′	
*Cytochrome b (mt-cytb)*	NC_005089	F: 5′-ACGCCATTCTACGCTCTATC-3′	95 bp
		R: 5′-GCTTCGTTGCTTTGAGGTGT-3′	
*MuRF1 (Trim63)*	NM_001039048	F: 5′-ACCTGCTGGTGGAAAACATC-3′	96 bp
		R: 5′-CTTCGTGTTCCTTGCACATC-3′	
*MUSA1 (fbxo30)*	NM_001168297, NM_027968	F: 5′-TCGTGGAATGGTAATCTTGC-3′	191 bp
		R: 5′-CCTCCCGTTTCTCTATCACG-3′	
*p62 (Sqstm1)*	NM_011018	F: 5′-GAAGCTGCCCTATACCCACA-3′	85 bp
		R: 5′-AGAAACCCATGGACAGCATC-3′	
*RNaseP (Rpp30)*	NM_019428	F: 5′-GAAGGCTCTGCGCGGACTCG-3′	100 bp
		R: 5′-CGAGAGACCGGAATGGGGCCT-3′	
*36b4 (Rplp0)*	NM_007475	F: 5′-AGGATATGGGATTCGGTCTCTTC-3′	143 bp
		R: 5′-TCATCCTGCTTAAGTGAACAAACT-3′	

F: forward, R: reverse.

Mitochondrial DNA (mtDNA) from muscle tissue samples was quantified as described with slight modifications [45]. Briefly, total DNA was extracted with the QIAamp DNA mini kit (Qiagen, Milano, Italy). Twenty ng of total DNA was assessed by qPCR. RNaseP gene was used as an endogenous control for nuclear DNA and the cytochrome b gene as a marker for mtDNA. Primer sequences are shown in Table 1.

### *In vivo* imaging using two-photon confocal microscopy

Mitochondrial morphology and autophagosome formation in living animals were monitored in *tibialis anterior* muscles transfected by electroporation with plasmids encoding pDsRed2-Mito or the LC3 protein fused to the yellow fluorescent protein (YFP-LC3), as described previously [29,38,46]. Two-photon confocal microscopy in the live, anaesthetised animals was then performed 12 days later on *in situ* exposure of transfected muscles [29,38,46]. To allow the muscle to recover from the injection-induced swelling, microscopic observation was interrupted for two to five minutes.

### Transmission electron microscopy

*Tibialis anterior* muscles were dissected and fixed for one hour in a solution containing 4% paraformaldehyde and 0.5% glutaraldehyde in 0.1 M cacodylate buffer, pH 7.4, immobilised on a Nunc Sylgard coated Petri dish (ThermoFisher Scientific, Waltham, MA, USA) to prevent muscular contraction as previously described [47]. The muscles were rinsed in the same buffer and dissected further into small blocks that were subsequently processed for transmission electron microscopy (TEM) as described elsewhere [48]. Briefly, the samples were postfixed with osmium tetroxide (2% in cacodylate buffer), rinsed, en bloc stained with 1% uranyl acetate in 20% ethanol, dehydrated and embedded in epoxy resin (Epon 812; Electron Microscopy Science, Hatfield, PA, USA) that was baked for 48 hours at 67°C. Thin sections were obtained with a Leica ultramicrotome (Reichert Ultracut E and UC7; Leica Microsystems, Wetzlar, Germany) stained with uranyl acetate and lead citrate, and finally examined with a Philips CM10 TEM (Philips, Eindhoven, The Netherlands). Morphometric analysis of mitochondrial cristae complexity was evaluated with a stereological method. Briefly, a regular grid has been superimposed over 10500X TEM micrographs and the number of intersections between the grid and mitochondrial cristae was recorded. The same grid was used for all the different analysis.

### Protein isolation and western blotting

Satellite cells were harvested and homogenised for 10 minutes at 4°C in RIPA lysis buffer, containing 50 mM Tris-HCl (pH 7.4), 150 mM NaCl, 1% NP-40, 1% sodium deoxycholate, 1 mM EDTA and 0.1% sodium dodecyl sulphate (SDS). Tissue samples from muscles were homogenised in a lysis buffer containing 20 mM Tris-HCl (pH 7.4), 150 mM NaCl, 1% Triton X-100, 10% glycerol, 10 mM EGTA and 2% SDS. Buffers were supplemented with a cocktail of protease and phosphatase inhibitors (cOmplete and PhosSTOP; Roche). Protein concentration was determined using the bicinchoninic acid assay (ThermoFisher Scientific). Using published protocols [49], SDS and β-mercaptoethanol were added to samples before boiling, and equal amounts of proteins (40 μg/lane) were separated by 4% to 20% SDS-polyacrylamide gel electrophoresis (Criterion TGX Stain-free precast gels and Criterion Cell system; Bio-Rad). Proteins were then transferred onto a nitrocellulose membrane using a Bio-Rad Trans-Blot Turbo System. The membranes were probed using the following primary antibodies as indicated in the text: goat polyclonal anti-HSP60 (N-20) and rabbit polyclonal anti-MyoD (C-20) (Santa Cruz Biotechnology, Dallas, TX, USA), mouse monoclonal anti-ClpP and rabbit polyclonal anti-LC3B (Sigma-Aldrich, Saint Louis, MO, USA), rabbit polyclonal anti-Mul-1 (Abcam, Cambridge, UK), mouse monoclonal anti-sarcomeric myosin (MF20) (Developmental Studies Hybridoma Bank, Iowa City, IA, USA), rabbit polyclonal anti-phospho-FoxO3a (Ser253), rabbit polyclonal anti-phospho-S6 ribosomal protein (Ser240/244), rabbit monoclonal anti-phospho-4E-BP1 (Thr37/46) (263B4) and rabbit polyclonal anti-phospho-Akt (Ser473) (Cell Signaling Technology, Danvers, MA, USA). After the incubation with the appropriate horseradish-peroxidase-conjugated secondary antibody (Cell Signaling Technology), bands were visualised using the Bio-Rad Clarity Western ECL substrate with a Bio-Rad ChemiDoc MP imaging system. To monitor for potential artefacts in loading and transfer among samples in different lanes, the blots were routinely treated with the Restore Western Blot Stripping Buffer (ThermoFisher Scientific) and reprobed with rabbit polyclonal anti-calnexin (GeneTex, Irvine, CA, USA), goat polyclonal anti-actin (I-19) or rabbit polyclonal anti-GAPDH (FL-335) primary antibodies (Santa Cruz Biotechnology). When appropriate, rabbit polyclonal anti-FoxO3a (75D8), rabbit monoclonal S6 ribosomal protein (54D2), rabbit polyclonal 4E-BP1 (53H11), and rabbit polyclonal Akt primary antibodies (Cell Signaling Technology) that recognise the protein independently of its phosphorylation state were also used in reprobing experiments.

### Confocal microscopy of myogenic precursor cells

Cells were plated in eight-well Nunc LabTeck Chamber slides (ThermoFisher Scientific). When indicated cells were transfected with YFP-LC3 plasmid. Transfections were performed with the Lipofectamine LTX with Plus reagent (Invitrogen-Life Technologies) according to the manufacturer’s instructions. The cells were used 24 hours after transfection in the various experimental settings described. For confocal imaging, the cells were fixed in paraformaldehyde and washed in phosphate-buffered saline [50]. To prevent nonspecific background, cells were incubated in 10% goat serum/phosphate-buffered saline followed by probing with the primary antibody mouse monoclonal anti-cyclophillin D (Abcam). Cells were then incubated with the secondary antibody, Alexa Fluor 546 dye-conjugated anti-mouse IgG (Molecular Probes-Life Technologies, Monza, Italy). Slides were placed on the stage of a TCS SP2 Laser-Scanning Confocal microscope (Leica Microsystems) equipped with an electronically controlled and freely definable Acousto-Optical Beam Splitter. Images were acquired with x63 magnification oil-immersion lenses. Analyses were performed using Imagetool software (Health Science Center, University of Texas, San Antonio, TX, USA). Images of cells expressing YFP-LC3 were thresholded by using the automatic threshold function.

### Immunohistochemistry and histology

Laminin and haematoxylin and eosin (H & E) staining were performed as previously described [47,51]. To measure the cross sectional area (CSA) of myofibres, muscle sections were stained with an anti-laminin A antibody (L1293; Sigma-Aldrich). Laminin, a cell-adhesion molecule strongly expressed in the basement membrane of skeletal muscle, was detected using an appropriate secondary antibody. Morphometric analyses were performed on sections collected from similar regions of each muscle using a Leica DMI4000 B automated inverted microscope equipped with a DCF310 digital camera. Image acquisition was controlled by the Leica LAS AF software. The ImageJ software was used to determine the CSA of 1,000 to 3,000 individual fibres from at least two different fields for each muscle section. Four to nine sections from each muscle were analysed. For histological analyses, serial muscle sections were obtained and stained in H & E following standard procedures. The number of fibres was counted and analysed using the ImageJ software.

Single myofiber isolation of hind limb muscle and nuclei immunofluorescence on single fibers was performed as previously described [8]. Nuclei of 30 individual fibres from each muscle were analysed.

### Whole body tension

The whole body tension (WBT) procedure was used to determine the ability of mice to exert tension in a forward pulling manoeuvre that is elicited by stroking the tail of the mice [52]. The tails were connected to a Grass FT03 transducer (Astro-Med, West Warwick, RI, USA) with a 4.0 silk thread (one end of the thread being tied to the tail and the other end to the transducer) [47]. Each mouse was placed into a small tube constructed of a metal screen with a grid spacing of 2 mm. The mice entered the apparatus and exerted a small resting tension on the transducer. Forward pulling movements were elicited by a standardised stroke of the tail with serrated forceps, and the corresponding forward pulling tensions were recorded using a Grass Polyview recording system (Astro-Med). Between 20 and 30 strokes of the tail forward pulling tensions were generally recorded during each session. The WBT was determined by dividing the average of the top ten or top five forward pulling tensions, respectively, by the body weight and represent the maximum phasic tension that can be developed over several attempts [52]. It is important to note that treatments or conditions which primarily alter muscle mass without changing the tension developed per unit of muscle mass produce corresponding alterations in forward pulling tension that are not associated with changes in either WBT 5 or WBT 10 [52,53].

### Treadmill running

Animals were made to run on a standard treadmill machine (Columbus Instruments, Columbus, OH, USA) either on a 0% grade or tilted 10% downhill starting at a warm-up speed of 5 m/minute for five minutes [54]. Every subsequent five minutes, the speed was increased by 5 m/minute until the mice were exhausted. Exhaustion was defined as the inability of the animal to return to running within 10 seconds after direct contact on an electric stimulus grid. Running time was measured and running distance calculated. Distance is the product of time and speed of the treadmill.

As a measure of membrane permeability, the Evans blue dye (EBD) assay was used [47]. A concentration of 5 μg/μl EBD prepared in physiological saline was injected intravenously through the tail vein. Injections (50 μl/10 g body weight) were performed 20 to 30 minutes after treadmill running. Mice were sacrificed 24 hours after EBD injection. *tibialis anterior* muscle sections (20 to 30 from each muscle) were then collected and the immunofluorescence of EBD-positive fibres was imaged using Texas red red filter. Creatine kinase (CK) serum levels (units per litre) were measured in blood samples obtained from the tail vein of mice after treadmill running. The blood was centrifuged at 13,000 × *g* at 4°C and the supernatant used to measure CK activity in an indirect colorimetric assay (Randox Laboratories, Crumlin, Northern Ireland, UK) [16,18].

### Statistics

Upon verification of normal distribution, the statistical significance of the raw data between the groups in each experiment was evaluated using the unpaired Student’s *t*-test (single comparisons) or one way analysis of variance (ANOVA) followed by the Newman-Keuls post-test (multiple comparisons). The GraphPad Prism software package (GraphPad Software, La Jolla, CA, USA) was used. After statistics (raw data), data from different experiments were represented and averaged in the same graph. The results are expressed as means ± SEM of the indicated n values.

### Chemicals

pDsRed2-Mito was a gift of Prof. Luca Scorrano (University of Padova, Padova, Italy). Dispase was purchased from Gibco-Life Technologies (Monza, Italy). TMRM and the secondary antibody for laminin experiments were obtained from Molecular Probes-Life Technologies. Iscove’s modified Dulbecco’s medium, penicillin, streptomycin, gentamycin, horse serum, and foetal bovine serum were purchased from Euroclone (Pero, Italy). Matrigel was obtained from BD-Bioscience (Milano, Italy). Primer pairs were obtained from Primmbiotech (Milano, Italy). Fibroblast growth factor was purchased from Tebu-bio (Milano, Italy). DETA-NO and KT5823 were obtained from Merck Millipore (Darmstadt, Germany). ODQ and cyclosporine were purchased from Enzo Life Sciences (Farmingdale, NY, USA). L^ω^-arginine methylester (L-NAME) and the other chemicals were purchased from Sigma-Aldrich.

## Results

### nNOSμ deficiency leads to mitochondrial dysfunction

**Figure 1:**
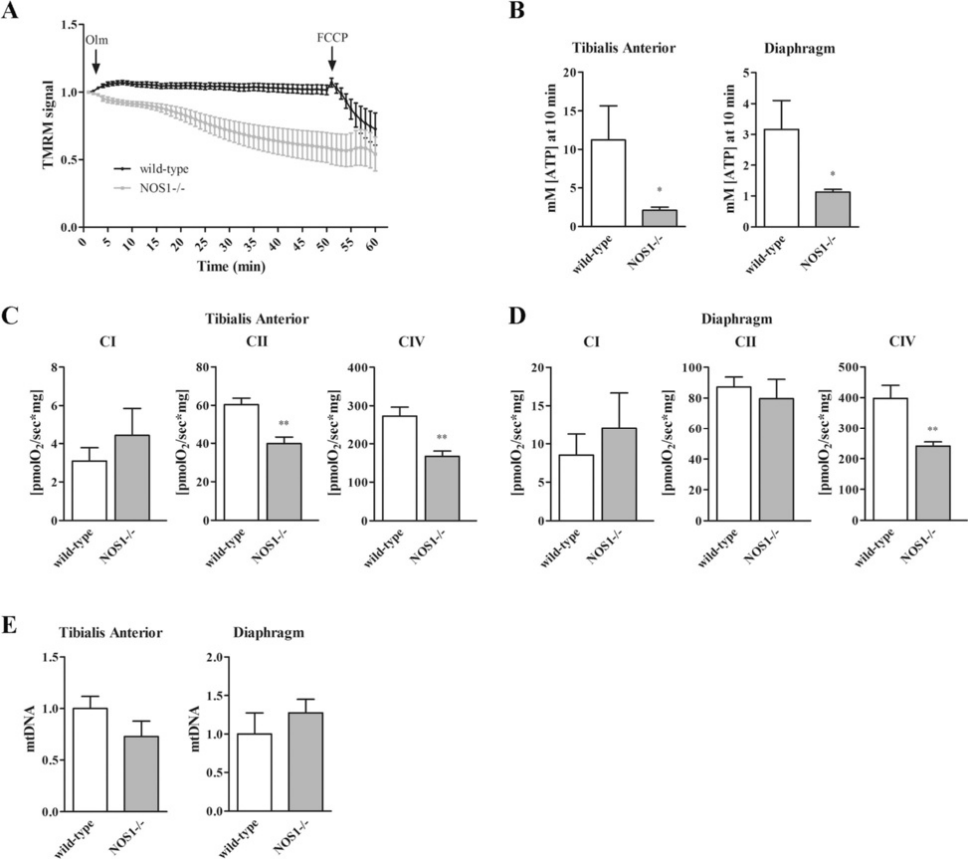
**Mitochondrial metabolism is impaired in skeletal muscles of NOS1-/- mice.** Fibres were isolated from different muscles of wild-type and NOS1-/- mice at P120. **(A)** Mitochondrial membrane potential measured in fibres isolated from *flexor digitorum brevis* muscles, loaded with TMRM and treated with 5 μM oligomycin (Olm) or 4 μM FCCP. TMRM staining was monitored in six to ten fibres obtained from at least three different animals per experimental group. Data are expressed by setting the initial value as 1. **(B)** ATP production on mitochondria isolated from *tibialis anterior* and diaphragm muscles, at 10 minutes after substrate addition. Data are expressed by setting the initial value as 1. **(C-D)** Oxygen consumption on fibres isolated from *tibialis anterior* and diaphragm muscles, supplied with specific CI, CII and CIV mitochondrial complex substrates, as indicated in the Methods. **(E)** Quantitative analysis of the mtDNA copy number. Data are expressed by normalizing mtDNA values versus nuclear DNA. Each histogram represents the data obtained from at least five different animals per experimental group. * *P* <0.05 and ** *P* <0.01 versus the respective wild-type control.

Mitochondrial function in skeletal muscles of adult NOS1-/- mice, that is, at P120, was dissected and compared with that of the respective age-matched wild-type littermattes (control). Mitochondrial membrane potential was monitored in isolated fibres from *flexor digitorum brevis* muscles loaded with TMRM, a potentiometric fluorescent dye. TMRM accumulates in the mitochondria that maintain a polarised mitochondrial membrane potential. A latent mitochondrial dysfunction masked by the ATP synthase operating in a reverse mode, that is, to consume ATP in order to maintain the mitochondrial membrane potential, can be unveiled using the ATP synthase inhibitor oligomycin [25]. In agreement with previous reports [29], addition of oligomycin to control mice fibres did not cause immediate changes in membrane potential even after extensive incubation (Figure 1A). Conversely, mitochondria in fibres of NOS1-/- mice underwent marked depolarisation after oligomycin.

We investigated whether the latent mitochondrial dysfunction observed in muscles of NOS1-/- mice affected the muscle bioenergetic parameters. To this end, we measured ATP generation from OXPHOS in isolated mitochondria of *tibialis anterior* and diaphragm muscle fibres. As shown in Figure 1B, total OXPHOS-generated ATP was significantly lower in NOS1-/- mice when compared to control.

We then analysed the mitochondrial bioenergetics in intact fibres using an *in situ* approach measuring oxygen consumption by high resolution respirometry. By this approach, we found that the maximal tissue mass-specific OXPHOS capacity with physiological combinations of CI mitochondrial complex substrates was similar in both *tibialis anterior* and diaphragm of NOS1-/- and control mice (Figure 1C-D). In contrast, the CII-linked respiratory capacity in *tibialis anterior* of NOS1-/- mice was lower than that in control muscle fibres, while no difference was observed in the diaphragm. In both *tibialis anterior* and diaphragm of NOS1-/- mice the CIV-linked respiratory capacity decreased significantly with respect to the controls. Of interest, qPCR analysis of mtDNA levels in *tibialis anterior* and diaphragm muscles did not reveal any difference between NOS1-/- and control mice (Figure 1E) suggesting that mitochondrial mass was not affected and defects in OXPHOS were due to dysfunctional mitochondria.

### nNOSμ deficiency affects mitochondrial network remodelling, UPR^mt^ and autophagy

**Figure 2:**
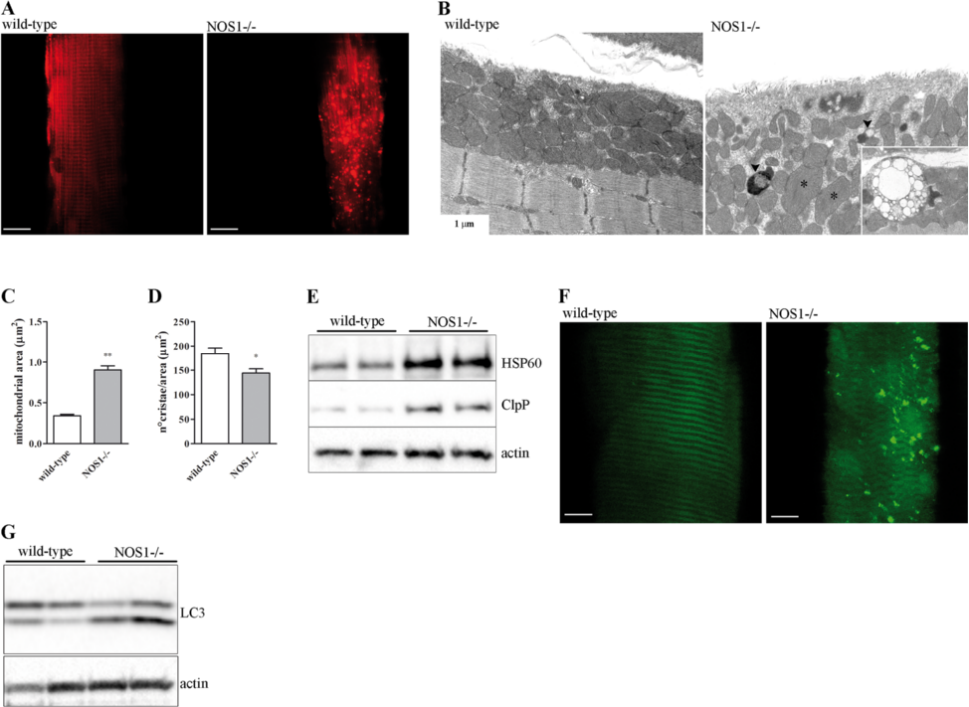
**Mitochondrial morphology, UPR** ^**mt**^ **and autophagy in skeletal muscles of NOS1-/- mice.** *Tibialis anterior* muscles were isolated from wild-type and NOS1-/- mice at P120. **(A)** *In vivo* imaging of the mitochondrial network by two-photon confocal microscopy. Muscles were transfected with the mitochondrially targeted red fluorescent protein pDsRed2-Mito. The images are representative of results obtained from at least five different animals per experimental group. Scale bar: 10 μm. **(B)** TEM images detecting the presence of abnormal, enlarged subsarcolemmal mitochondria (asterisks) or autophagic vacuoles (arrowheads) in NOS1-/- muscles. The inset depicts a multivesicular body in NOS1-/- fibres taken at higher magnification. The images are representative of results obtained from at least three different animals per experimental group. **(C-D)** Subsarcolemmal mitochondrial ultrastructure analysis by TEM. Data represent the quantification of the mitochondrial area and morphometric analysis of mitochondrial cristae complexity. Each histogram represents the data obtained from at least three different animals per experimental group. * *P* <0.05 and ** *P* <0.01 versus the respective wild-type control. **(E)** Western blot analysis of HSP60 and ClpP expression. Actin was used as the internal standard. The image is representative of results obtained from at least five to seven different animals per experimental group. **(F)** *In vivo* imaging of autophagosome formation by two-photon confocal microscopy. Muscles were transfected with YFP-LC3. The images are representative of results obtained from at least five different animals per experimental group. Scale bar: 10 μm. **(G)** Western blot analysis of LC3 lipidation. Actin was used as the internal standard. The image is representative of results obtained from at least 10 different animals per experimental group.

Alterations in the content, shape or function of the mitochondria have been associated with muscle homeostasis [31,55]. To identify the changes in mitochondrial network morphology, *tibialis anterior* muscles of P120 NOS1-/- and wild-type control mice were imaged using pDsRed2-Mito, a mitochondrially targeted red fluorescent protein, by *in situ* two-photon confocal microscopy [29,38,46]. NOS1-/- mice showed a disorganised mitochondrial network (Figure 2A). Accordingly, ultrastructural analyses by TEM (Figure 2B and Additional file 1: Figure S1A) revealed changes in the subsarcolemmal mitochondria of *tibialis anterior* muscles of NOS1-/- mice that exhibited, in thin sections, a significant increase in mitochondrial surface area (Figure 2C) and a significant decrease in the density of the *cristae* (Figure 2D), as compared with the controls. The same evaluation was performed on subsarcolemmal mitochondria from diaphragm muscle with similar results (data not shown). The analysis of intermyofibrillar mitochondria (see Additional file 1: Figure S1B) showed a pattern of enlarged mitochondria indicating that the presence of these mitochondrial alterations in NOS1-/- mice muscle is not restricted to the sarcolemma but is a more general phenomenon.

We then analysed two downstream processes linked to mitochondrial stress: UPR^mt^ and autophagy. In *tibialis anterior* muscles of P120 NOS1-/- mice the expression of the nuclearly-encoded mitochondrial chaperones HSP60 and the protease ClpP, which correlates with the level of unfolded proteins in mitochondria [56,57], was found to be higher than in the controls (Figure 2E). In addition, the two-photon confocal microscopy of the YFP-LC3 [29,38,46] revealed the presence of LC3-positive vesicles, an established marker of autophagosome formation [58], in *tibialis anterior* muscles of P120 NOS1-/- mice (Figure 2F). Furthermore, TEM analysis showed the presence of autophagic vacuoles and multivesicular bodies, indicative of an active autophagic pathway [59], in *tibialis anterior* and diaphragm muscles of P120 NOS1-/- mice (Figure 2B and Additional file 1: Figure S1C). The enhanced autophagy in the absence of nNOSμ in skeletal muscle was confirmed by Western blot analysis. The appearance of a faster migrating band of LC3 protein due to its lipidation and cleavage is a common marker of autophagy induction [58]. As shown in Figure 2G, *tibialis anterior* muscles of P120 NOS1-/- mice exhibited increased lipidated LC3 levels when compared to control mice. Similar results on LC3 conversion were obtained analysing diaphragm muscle samples (see Additional file 1: Figure S1D).

### NO signalling regulates UPR^mt^ and autophagy machinery

**Figure 3:**
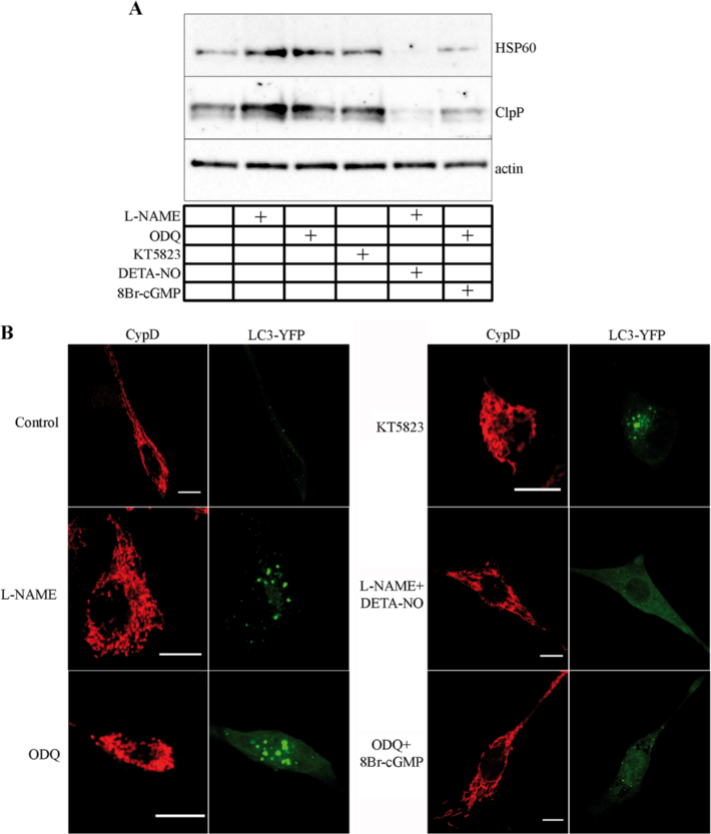
**NO signalling, UPR** ^**mt**^ **, and autophagy on myogenic precursor cells.** Cells were differentiated for six hours in the absence (control) or in the presence of L-NAME (6 mM), ODQ (10 μM), KT5823 (1 μM), L-NAME + DETA-NO (80 μM), and ODQ +8 Br-cGMP (2.5 mM). **(A)** Western blot analysis of HSP60 and ClpP expression. Actin was used as the internal standard. **(B)** Confocal microscopy imaging of cells transfected with YFP-LC3. Mitochondrial morphology was detected by mitochondrial matrix-specific protein cyclophillin D (CypD) staining. Scale Bar: 10 μm. Images are representative of at least three to five independent experiments..

Activation of the NO-dependent enzyme guanylate cyclase, with formation of cyclic guanosine monophosphate (cGMP) and activation of a variety of downstream signalling cascades, including cGMP-dependent-protein kinases (PKG), contributes significantly to mediate the physiological effects of NO in muscle [2,5,60]. To investigate the involvement of the cGMP-dependent signalling on UPR^mt^ and autophagy, myogenic precursor cells were differentiated for six hours in the absence (control) or in the presence of the inhibitor of NOS L-NAME (6 mM), the inhibitor of guanylate cyclase ODQ (10 μM), and the inhibitor of PKG KT5823 (1 μM) [61-66]. L-NAME, ODQ and KT5823 treatment increased the expression of HSP60 and ClpP protein. The NO donor DETA-NO (80 μM) and the membrane-permeant cGMP analogue 8Br-cGMP (2.5 mM) [62-66] reversed the effects of L-NAME and ODQ, respectively (Figure 3A).

In another set of experiments, cells were transiently transfected with YFP-LC3 and then differentiated. As shown by confocal microscopy fluorescence analysis of LC3 and the mitochondrial matrix-specific protein cyclophillin D (Figure 3B), in control cells LC3 staining was diffuse and the majority of mitochondria were in the elongated form, indicating myogenic differentiation [25] and a low rate of autophagy. L-NAME, ODQ, and KT5823 treatment, while inducing mitochondrial fragmentation, resulted in LC3 localisation into dot cytoplasmic structures, as compared to the diffuse cytoplasmic distribution observed in control cells. The effects of L-NAME and ODQ were prevented by DETA-NO and 8Br-cGMP, respectively.

**Figure 4:**
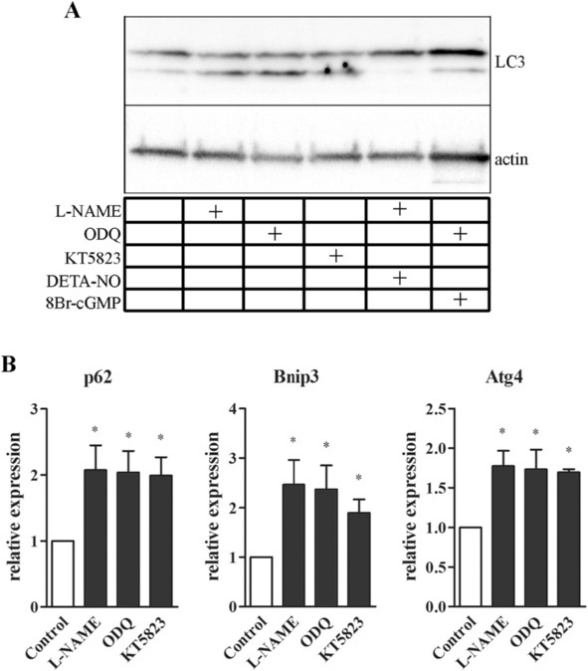
**NO signalling and autophagic pathway on myogenic precursor cells. (A)** Western blot analysis of LC3 lipidation in cells differentiated for six hours in the absence or in the presence of L-NAME (6 mM), ODQ (10 μM), KT5823 (1 μM), L-NAME + DETA-NO (80 μM), and ODQ +8 Br-cGMP (2.5 mM). Actin was used as the internal standard. Image is representative of at least five independent experiments. **(B)** qPCR analysis of mRNA levels for p62, Bnip3 and Atg4 in cells differentiated for six hours in the absence (control) or in the presence of L-NAME ODQ, and KT5823. Values are expressed as the fold change over control. Each histogram represents the data obtained from at least five independent experiments. * *P* <0.05 versus respective control.

NO control of autophagy was assessed further by analysing the expression of relevant markers of the autophagic signalling pathway, namely LC3, by western blotting and p62, Bnip3 and Atg4 by qPCR analysis [58,67]. L-NAME, ODQ and KT5823 treatments increased lipidated LC3 conversion in differentiated satellite cells and LC3 lipidation induced by L-NAME and ODQ was blocked by DETA-NO or 8Br-cGMP, respectively (Figure 4A). In addition, cells treated with L-NAME, ODQ and KT5823 expressed higher levels of transcripts encoding p62, Bnip3 and Atg4 (Figure 4B).

### Deficient nitric oxide signalling promotes FoxO3-Mul-1 axis

**Figure 5:**
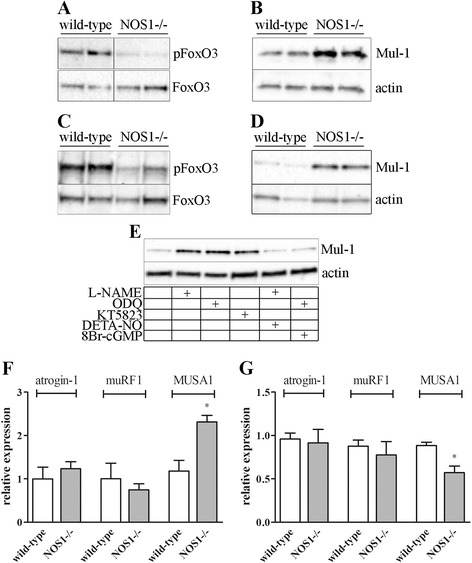
**NO signalling, FoxO3, and ubiquitin ligases.** Western blot analysis of phosphorylated FoxO3 levels (pFoxO3) or mitochondrial ubiquitin ligase Mul-1 expression in *tibialis anterior* **(A-B)** and diaphragm **(C-D)** of wild-type and NOS1-/- mice at P120. FoxO3 or actin were used as the internal standard. The images are representative of results obtained from at least four to ten different animals per experimental group. **(E)** Western blot analysis of Mul-1 expression in myogenic precursor cells differentiated in the absence or in the presence of L-NAME (6 mM), ODQ (10 μM), KT5823 (1 μM), L-NAME + DETA-NO (80 μM) and ODQ +8 Br-cGMP (2.5 mM). Actin was used as the internal standard. The image is representative of at least five independent experiments. qPCR analysis of mRNA levels for atrogin-1, muRF1 and MUSA1 in *tibialis anterior* **(F)** and diaphragm **(G)** muscles of wild-type and NOS1-/- mice at P120. Values are expressed as the fold change over wild-type. Each histogram represents the data obtained from at least five to eight different animals per experimental group. * *P* <0.05 versus the respective wild-type control.

Catabolic conditions activate FoxO transcription factors, which stimulate the ubiquitin-proteasome system as a response to skeletal muscle-wasting [31,55]. FoxO3 activity is necessary and sufficient for the induction of autophagy in skeletal muscle [38]. FoxO3 translocation from the cytoplasm to the nucleus determines the direct transcriptional activation of genes essential to autophagosome formation, namely p62, Bnip3 and Atg4 [58,67]. Enhanced activity of FoxO transcription factors has also been associated with disruption of mitochondrial function and organisation leading to impaired skeletal muscle function and development [29]. As shown in Figure 5A, C, phosphorylated FoxO3 levels in *tibialis anterior* and diaphragm muscles of P120 NOS1-/- mice were lower than in the controls. In addition, *tibialis anterior* and diaphragm muscles of NOS1-/- mice overexpressed the protein corresponding to mitochondrial ubiquitin ligase Mul-1 (Figure 5B, D), which has been recently reported to be upregulated in muscle through FoxO3 transcription factors and promoting mitochondrial fission, depolarization and mitophagy [68,69]. As shown in Figure 5E, *in vitro* treatment of differentiated myogenic precursor cells from wild-type control mice with L-NAME, ODQ and KT5823 increased Mul-1 protein expression. The effects induced by L-NAME and ODQ were blocked by DETA-NO and 8Br-cGMP, respectively. In *tibialis anterior* and diaphragm muscles of P120 NOS1-/- mice, qPCR analysis of other E3 ubiquitin ligases, atrogin-1 and MuRF1, involved in muscle loss [69,70], ruled out a nNOSμ-dependent modulation of their expression (Figure 5F, G). Also, the differences obtained with MUSA1 analysis are difficult to correlate with nNOSμ deficiency. These findings indicate that the effects of nNOSμ absence on E3 ubiquitin ligases mainly affect expression of Mul-1 gene.

### nNOSμ deficiency affects muscle growth

**Figure 6:**
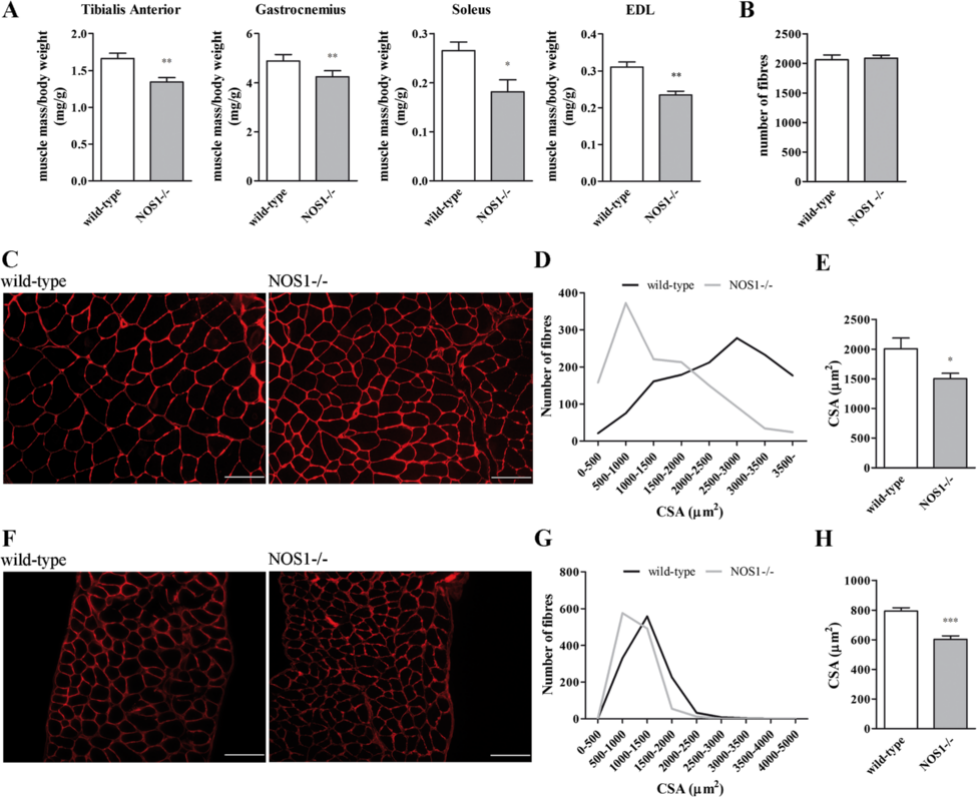
**Skeletal muscle phenotype of wild-type and NOS1-/- mice at P120. (A)** Weight of *tibialis anterior*, *gastrocnemius*, *soleus*, and *extensor digitorum longus* (EDL) muscles. The muscle size is relative to body weight. Each histogram represents the data obtained from at least 10 different animals per experimental group. **(B)** The number of myofibres in *tibialis anterior.* Each histogram represents the data obtained from at least four to five different animals per experimental group. Laminin staining of *tibialis anterior* **(C-E)** and diaphragm **(F-H)** muscles. **(C, F)** Immunohistochemical images. Scale bar: 100 μm. **(D, G)** Representative distribution of CSA values. **(E, H)** Quantification of CSA. Images and quantifications represent the data obtained from at least four to seven different animals per experimental group. *P <0.05, **P <0.01, and ***P <0.001 versus the respective wild-type control.

We evaluated the effects of the absence of nNOSμ on skeletal muscle phenotype. *Tibialis anterior*, *gastrocnemius*, *soleus*, and *extensor digitorum longus* muscles were dissected and weighed. Since the body weight and the visceral adipose tissue of NOS1-/- male mice were significantly lower than wild-type control (see Additional file 2: Figure S2A-B) [71] we calculated the muscle size relative to body weight [72]. As shown in Figure 6A and Additional file 2: Figure S2C, the relative mass of the muscles for the P120 NOS1-/- mice was significantly lower than the relative mass of the muscles for the control mice. This excludes the possibility that the changes in muscle mass are simply due to an overall change in size of the mice.

The overall morphology of the *tibialis anterior* and diaphragm muscle in P120 NOS1-/- mice was normal, without pathological features of necrosis, macrophage infiltration and centronucleated fibres (see Additional file 2: Figure S2D). In addition, the number of fibres in *tibialis anterior* muscles was comparable in both NOS1-/- and control mice (Figure 6B). By contrast, laminin staining of *tibialis anterior* and diaphragm, used to identify individual muscle fibres, revealed a significant decrease in the mean CSA of *tibialis anterior* and diaphragm sections in P120 NOS1-/- mice when compared with control (Figure 6C-H).

**Figure 7:**
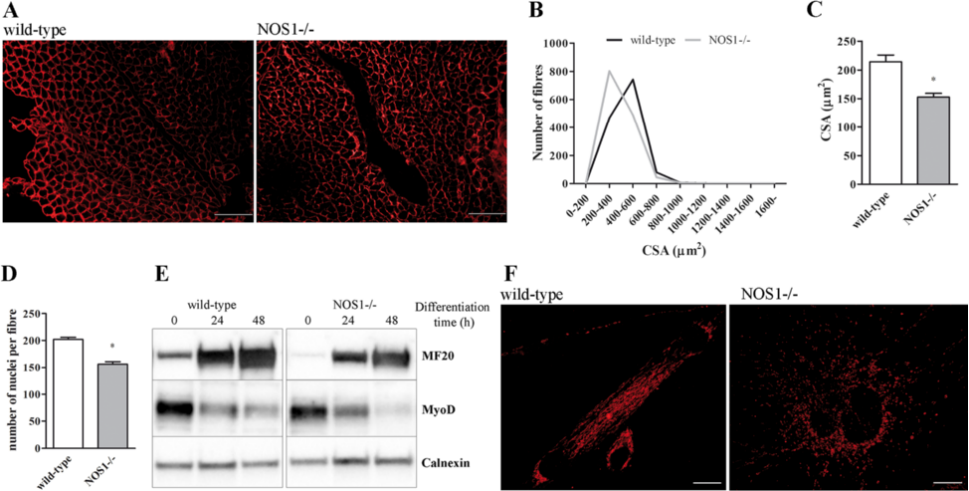
**Skeletal muscle phenotype of wild-type and NOS1-/- mice at P10. (A-C)** Laminin staining of hind limb muscles. **(A)** Immunohistochemical images. Scale bar: 100 μm. **(B)** Representative distribution of CSA values. **(C)** Quantification of CSA. Images and quantifications represent the data obtained from at least five different animals per experimental group. **(D)** Number of myonuclei per fibre in hind limb muscles. Each histogram represents the data obtained from at least three different animals per experimental group. **(E)** Western blot analysis of myosin (MF20) and MyoD expression in myogenic precursor cells isolated from wild-type and NOS1-/- mice and differentiated for increasing times. Calnexin was used as the internal standard. Images are representative of at least three independent experiments. **(F)** Confocal microscopy imaging of myogenic precursor cells isolated from wild-type and NOS1-/- mice and differentiated for 48 hours. Mitochondrial morphology was detected by mitochondrial matrix-specific protein cyclophillin D staining. Scale Bar: 10 μm. Images are representative of at least three independent experiments. * *P* <0.05 versus the respective wild-type control.

The examination of multiple time points was then carried out in order to establish a possible link between the changes in mitochondrial homeostasis and the reduction in muscle size. The CSA (Figure 7A-C) and the number of myonuclei (Figure 7D) of hind limb muscle fibres were significantly decreased in P10 NOS1-/- mice, when compared with the respective control. Muscle growth during post-natal development (P0 to P21), but not at later stages, is accompanied by a continuous increase in the number of myonuclei resulting from satellite cell fusion [69,73]. As shown in Figure 7E, NOS1-/- cells exhibited lower levels of myosin and MyoD, which are markers of myogenic differentiation, as compared to control cells. Interestingly, CycloD staining of differentiating myogenic precursor cells indicated that the absence of nNOSμ induces diffuse mitochondrial fragmentation (Figure 7F) [25]. Taken together, our data argue that the absence of nNOSμ induces mitochondrial fragmentation and a deficit in satellite cell fusion/differentiation, thus impairing fibre growth.

**Figure 8:**
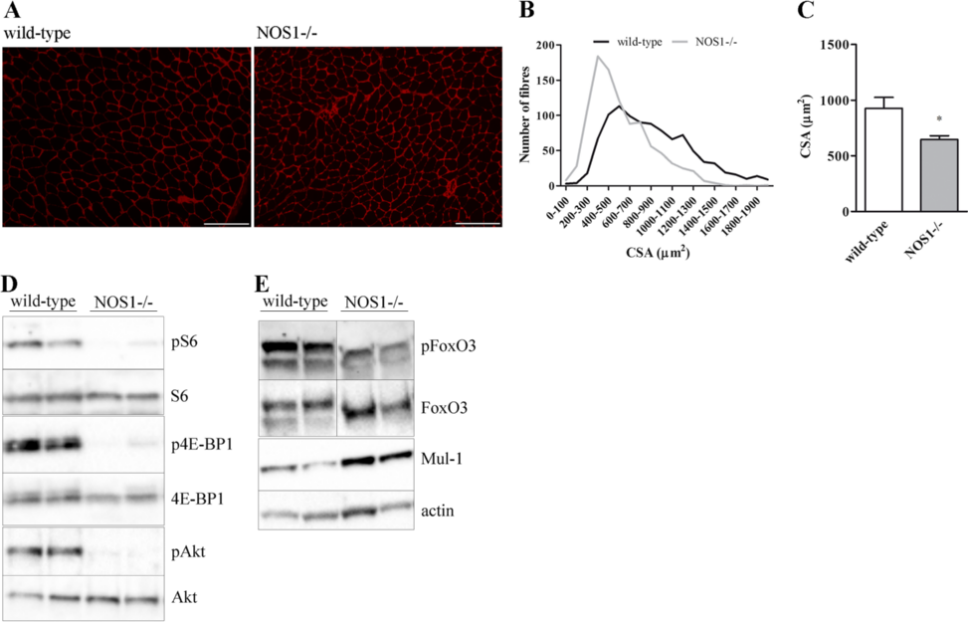
**Skeletal muscle phenotype of wild-type and NOS1-/- mice at P30. (A-C)** Laminin staining of *tibialis anterior* muscles. **(A)** Immunohistochemical images. Scale bar: 100 μm. **(B)** Representative distribution of CSA values. **(C)** Quantification of CSA. Images and quantifications represent the data obtained from at least five different animals per experimental group. Western blot analysis in *tibialis anterior*: **(D)** phosphorylated S6, 4E-BP1 and Akt levels, **(E)** phosphorylated FoxO3 levels or mitochondrial ubiquitin ligase Mul-1 expression. S6, 4E-BP1, Akt, FoxO3 or actin were used as the internal standard. The images are representative of results obtained from at least four different animals per experimental group. *P <0.05 versus the respective wild-type control.

At P30 we found that the CSA of *tibialis anterior* was significantly decreased in NOS1-/- mice, when compared with controls (Figure 8A-C). In this crucial time of muscle growth we also measured the activation of the Akt-mTOR pathway as a positive regulator [55,69,73,74]. As shown in Figure 8D, phosphorylated levels of S6 ribosomal protein, 4E-BP1 and Akt in *tibialis anterior* muscles of NOS1-/- mice were lower than in the controls. FoxO3 proteins are phosphorylated by Akt, which renders them inactive; this may explain why phosphorylated FoxO3 levels were found to be lower as well, while Mul-1 was overexpressed (Figure 8E). Of importance, both events are correlated with muscle mitochondrial dysfunction and growth [29,55,68,69,73,74].

Using NOS1-/- mice it has been previously shown that nNOS modulates the mechanism of disuse-induced atrophy via FoxO transcription factors [75]. Our observation that at P10, P30 (see Additional file 2: Figure S2E-F) and P120 (Figure 5E-F) NOS1-/- and control mice expressed similar levels of transcripts encoding the classical atrogenes atrogin-1 and MuRF1 [69,70,75], indicates that the atrophy pathways do not play a key role in the development of NOS1-/- muscles.

### nNOSμ deficiency affects muscle function

**Figure 9:**
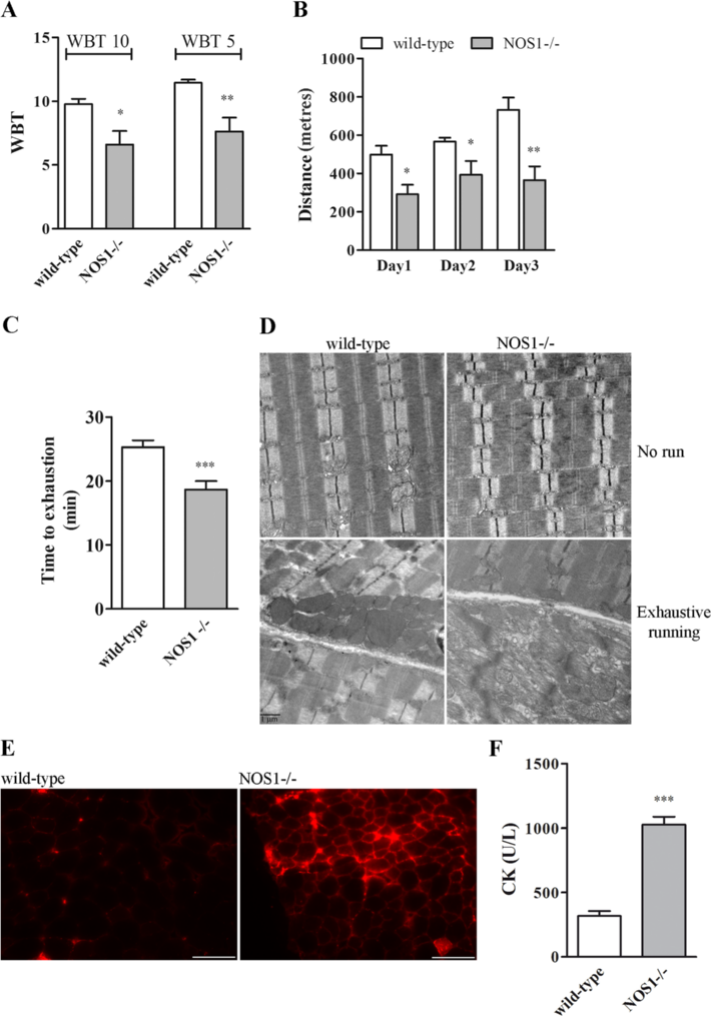
**Skeletal muscle function in wild-type and NOS1-/- mice. (A)** WBT measurements determined by dividing the average of the top ten or top five forward pulling tensions, respectively, by the body weight. **(B)** Running distance calculated during one bout of exhaustive treadmill running (day 1) and after repeated challenges (days 2 and 3). **(C)** Treadmill runtime to exhaustion calculated as the averages obtained at day 1 to 3. Each histogram represents the data obtained from at least four to five different animals per experimental group. **(D)** TEM analysis performed in *tibialis anterior* muscles of both unchallenged (no run) and challenged (exhaustive running) mice. The images are representative of results obtained from at least three different animals per experimental group. **(E)** EBD uptake in *tibialis anterior* muscles after the treadmill running. Scale Bar: 100 μm. The images are representative of results obtained from at least four different animals per experimental group. **(F)** CK serum levels (units per litre) of mice after treadmill running. Each histogram represents the data obtained from at least four different animals per experimental group. *P <0.05, **P <0.01 and ***P <0.001 versus the respective wild-type control. WBT and treadmill running were performed on animals at P120.

We evaluated whether the absence of nNOSμ affected skeletal muscle function. The WBT measurement determines the total phasic forward pulling tension exerted by the fore and hind limb muscles and reflects the maximal acute phasic force the mouse can achieve to escape a potentially harmful event [52]. As shown in Figure 9A, the WBT normalised for body weight in P120 NOS1-/- mice was significantly lower than in the wild-type control, consistent with an unpaired muscle specific force output in the absence of nNOSμ.

We also examined the muscle resistance to fatigue: we subjected NOS1-/- mice to treadmill running, that measures resistance to fatigue during a forced exercise, and examined both exercise performance and tolerance. As shown in Figure 9B, the total distance run by NOS1-/- mice during one bout of exhaustive treadmill running (day 1) was significantly lower when compared to controls. This reduction in performance of NOS1-/- mice was also observed after repeated challenges: NOS1-/- mice showed significant exercise intolerance after repetitive exercise challenges, while control mice at day 3 showed even improved exercise capacity, compared to day 1. NOS1-/- mice also exhibited a significantly decreased treadmill runtime to exhaustion (Figure 9C).

We then assessed the structure/damage of skeletal muscle myofibres after exercise. TEM analysis performed in *tibialis anterior* muscles of P120 NOS1-/- mice after the treadmill running showed marked ultrastructural changes, as, for instance, defects in the organisation of the contractile apparatus (sarcomere), that were observed neither in the wild-type mice nor in unchallenged NOS1-/- mice (Figure 9D). The features observed in challenged NOS1-/- mice might be a direct consequence of denervation events as also indicated by collagen fibres deposition and motor end-plates lacking the presynaptic nerve ending (data not shown). As shown in Figure 9E, *tibialis anterior* muscles of P120 NOS1-/- mice after the treadmill running displayed an increased uptake versus wild-type of EBD, which stains damaged myofibres [47]. As an *in vivo* indicator of skeletal muscle damage we also analysed the serum levels of CK, a skeletal muscle enzyme released during fibre degeneration whose activity increased in dystrophic animals [16,18]. As expected, in NOS1-/- mice after the treadmill running, the serum CK activity was found to be significantly higher than that in the wild-type mice (Figure 9F).

## Discussion

This study documents that nNOSμ deficiency, while severely altering the structure and bioenergetics potential of skeletal muscle mitochondria does not impact significantly on the overall resting muscle structure, apart from reducing muscle mass and the CSA of the myofibres of specific muscles. When the muscle is exposed to workloads, however, the consequences of nNOSμ deficiency become apparent, with a significantly reduced resistance of the muscles accompanied by increased sensitivity to exercise-induced damage. This establishes for the first time a link between a deficit in NO signalling, mitochondrial alterations and skeletal muscle impairments.

The first result emerging from our analysis is that nNOSμ deficiency is *per se* sufficient to induce profound defects in mitochondria, with alterations in mitochondrial distribution, shape, morphology and size accompanied by a latent mitochondrial dysfunction such that energy generation is impaired. Nitric oxide has several key functions in mitochondria: it inhibits mitochondrial fission, induces mitochondrial biogenesis and controls mitochondrial respiratory rate by reversible inhibition of complex IV in the mitochondrial respiratory chain [25,76,77]. Furthermore, it controls the expression of several enzymes in the Krebs cycle [78]. Derangement of these mitochondrial functions is most likely at the basis of the multiple mitochondrial deficits we observed in NOS1-/- mice.

Of importance, we found that this overall mitochondrial dysfunction was accompanied both in intact myofibres *in vivo* and in isolated satellite cells *in vitro* by an enhanced UPR^mt^ response. It has been hypothesised that the UPR^mt^ is activated prior to the induction of autophagy [79]; in particular, that the autophagy pathway is activated when mitochondria cannot maintain a polarised membrane potential despite UPR^mt^ activation. We found that the increase in UPR^mt^ was accompanied by autophagy and increased expression of molecules relevant to autophagic signalling, namely p62, Bnip3 and Atg4. This suggests that nNOSμ deficiency leads to a sufficiently severe mitochondrial deficit that cannot be restored by UPR^mt^. The enhanced autophagic and UPR^mt^ response were normalised when the cGMP-dependent signalling was activated, indicating that these events are controlled by NO *via* its physiological second messenger cGMP.

The second relevant information is that an altered NO system leads to impairment of muscle function that is selective to specific parameters and unmasked during exercise. In particular we found that skeletal muscles in the absence of nNOSμ are smaller relative to the rest of the body, thus indicating that muscle mass decrease was not simply attributable to a generalised decreased body mass tissues (including adipose tissue) and likely due to a specific reduction in the size of the muscle fibres themselves. In agreement with this, NOS1-/- mice muscles (that is, *tibialis anterior* and diaphragm) displayed smaller myofibre CSA when compared to littermate controls, although they did not show any pathological features reminiscent of muscle damage, such as inflammation, necrosis or fibrosis. Similar morphological data were obtained in male NOS1-/- mice backcrossed onto the B6129 background (our experimental model) [71] or backcrossed onto the C57BL/6 background [80], although in the latter model no difference in *tibialis anterior* muscle mass relative to body mass was reported. That the decrease in muscle mass is due to mechanisms other than the decrease in body mass was recently suggested using NOS1-/- *mdx* mice [72]. The deficiency of nNOSμ is also accompanied by muscle ageing [81] and fibre growth was prevented in the NOS1-/- mice model of skeletal muscle hypertrophy [82] and NOS1-/- *mdx* mice [72]. In a recent study, no difference in the weight and CSA of *tibialis anterior* muscles from NOS1-/- and control was also reported but the animal background was not indicated [83]. Discrepancies in these studies may be explained, at least in part, by strain-specific modulation of the nNOSμ-regulated phenotype, a hypothesis substantiated by the observation, by the same group, that morphological data differed between NOS1-/- mice backcrossed onto the C57BL/6 and the B6129 background [37,71,80].

The functional studies revealed two important aspects of the role of NO in skeletal muscle. Firstly, the fact that NOS1-/- mice in our *in vivo* experiments exhibited a deficit in forward pulling tension and resistance to fatigue during a forced exercise indicates that nNOSμ is important to maintain skeletal muscle strength and the animal’s ability to perform in repetitive exercise training. Our results *in vivo* are in line with a previous study with an *in situ* approach reporting that nNOSμ-deficient *tibialis anterior* muscles exhibit a reduced force production and a specific deficit in adapting to exercise and develop profound fatigue upon repeated contraction [71]. An excessive fatigue has been also observed in NOS1-/- mice and wild-type mice treated with a nNOS inhibitor [12]. A specific and intrinsic deficit in muscle force production has been recently reported in NOS1-/- *mdx* mice, although muscle fatigue was unaffected by nNOS depletion [72]. Secondly, our data on muscle phenotype and CK measurements after treadmill running indicate that nNOSμ deficiency induces muscle degeneration/damage post-exercise. This raises the possibility that nNOSμ-deprived muscles cannot activate protective responses. Accordingly, NOS1-/- *mdx* mice displayed increased susceptibility to eccentric contraction-induced muscle damage [72]. In addition, expression of a muscle-specific nNOS transgene prevents muscle membrane injury during modified muscle use [84]. In this respect, there is a general agreement that NO produced by nNOS plays an important role in muscle repair in chronic conditions [5,8,9] although the use of NOS1-/- mice suggested that nNOS is not essential to functional recovery after acute injury [80].

The third important observation is the correlation between mitochondrial defects and muscle impairment. Alterations in the content, shape or function of the mitochondria appear to occur in damaged muscle and inhibition of mitochondrial fission protects from muscle loss during fasting [29]. Recent findings have also underlined the crucial role of autophagy in the control of muscle mass and functions [29,31,55,69]. Autophagy derangement is involved in a number of inherited muscle diseases [31-33]. Of interest, mitochondria are involved in regulating autophagy [30]. In addition, skeletal muscle was shown to be sensitive to the physiological stressors that trigger the UPR^mt^ [35,36] and UPR^mt^ is activated in skeletal muscle during exercise as part of an adaptive response to exercise training [54]. Here, we raise the possibility that mitochondrial dysfunction, UPR^mt^ and autophagy are functionally related to each other and promoted by a single event, that is, the deficit in NO signalling, thus suggesting that the association of altered mitochondrial homeostasis and muscle phenotype/performance in NOS1-/- mice is not coincidental.

The experiments we carried-out in myogenic precursor cells and NOS1-/- mice during critical stages of muscle development are consistent with an association of altered mitochondrial homeostasis and muscle phenotype/performance and provide an indication of the mechanism responsible for the impaired fibre growth resulting in a deficit of muscle performance. In particular, nNOSμ absence altered mitochondrial homeostasis in myogenic precursor cells with a decrease in the number of myonuclei per fibres and impaired muscle development at early stages of growth. This also suggests that fusion of myogenic precursor cells during perinatal myogenesis is impaired. Accordingly, NO has been shown to stimulate the ability of myogenic precursor cells to become activated and fuse to each other [5,8,85]. There is a general agreement that mitochondria change when the myoblasts differentiate into myotubes [27]. Also, NO maintains functional mitochondria and this permits differentiation of myogenic precursor cells *in vitro* [25]. At the signalling level, the Akt-mTOR pathway and Akt-FoxO3-Mul-1 axis are involved in skeletal muscle growth/wasting, autophagy and mitochondrial dysfunction [29,31,38,46,55,58,67-69,73,74]. Of interest, Mul-1 has been recently reported to be upregulated during muscle wasting, possibly *via* an autophagic mechanism involving FoxO3 transcription factors [68]. Our data indicate the relevance of the above signalling pathways and that they are controlled by NO. We observed an inhibition of the Akt-mTOR pathway in the absence of nNOSμ. Concomitantly, the Akt-FoxO3-Mul-1 axis was also dysregulated. In addition, the inhibition of the nNOS/NO/cGMP/PKG system induced the transcriptional activity of FoxO3 and increased Mul-1 expression. These events are likely associated with nNOSμ-dependent impairments of muscle fibre growth.

We cannot exclude that failure of other NO-dependent action involving, for instance, the vascular system, may have contributed to the functional and structural defects we observed in skeletal muscle. *Extensor digitorum longus* of NOS1-/- mice revealed an altered capillary-to-fibre ratio but not changes in the capillary ultrastructure or the hemodynamics at basal conditions [86]. Noteworthy, NO generated by sarcolemmal nNOSμ normally acts as a paracrine signal that optimises blood flow in the working muscle [12,87,88] and the protective vasodilating action is impaired in the contracting muscles of NOS1-/- mice [12,89]. In this respect, the lack of this vasodilating action in NOS1-/- mice has been suggested to affect muscle performance [71]. Results obtained in NOS1-/- mice with different cardiac injuries indicated a protective role of nNOS, although an opposite effect cannot be excluded [90,91]. The deficit in exercise performance of NOS1-/- muscles may be the consequence, at least in part, of a decreased oxygen delivery following blood flow impairment.

## Conclusions

Muscle exercise performance is a complex physiological process that can occur by many different mechanisms and NO has long been described to be relevant among them [2]. Our study now suggests that the relevance of NO also resides in the fact that it regulates key homeostatic mechanisms in skeletal muscle, namely mitochondrial bioenergetics and network remodelling, UPR^mt^ and autophagy. Although NOS1-/- mice do not display the overt features of myopathies, such as muscle degeneration, reactive regeneration and replacement of muscle with fibroadipous tissue [92,93], we clearly show that alterations of the NO system significantly impair muscle fibre growth, thus resulting in a deficit of muscle force and the ability to sustain prolonged exercise. This aspect may explain why NO deficiency contributes to muscle impairment in degenerative disease of the muscle, such as muscular dystrophies.

## Additional files

Additional file 1: Figure S1. Mitochondrial ultrastructure and LC3 lipidation in skeletal muscles of wild-type and NOS1-/- mice. **(A)** TEM images of subsarcolemmal mitochondria of *tibialis anterior* muscles. Scale bar: 0.1 μm. **(B)** TEM images of intermyofibrillar mitochondria of *tibialis anterior* muscles. Scale bar: 1 μm. **(C)** TEM images of diaphragm muscles detecting the presence of autophagic vacuoles (arrowheads) in NOS1-/- fibres. TEM images are representative of results obtained from at least three different animals per experimental group. **(D)** Western blot analysis of LC3 lipidation in diaphragm muscles of wild-type and NOS1-/- mice. GAPDH was used as internal standard. The image is representative of results obtained from at least 10 different animals per experimental group. Analyses were performed on animals at P120. Additional file 2: Figure S2. Weight, muscle structure and muscle expression of ubiquitin ligases in wild-type and NOS1-/- mice. Body **(A)** and visceral adipose tissue (VAT) **(B)** weight. Each histogram represents the data obtained from at least three to eight different animals per experimental group. **P* <0.05, and ***P* <0.01 versus the respective wild-type control. **(C)** Pictures of *tibialis anterior*, *soleus*, and gastrocnemius muscles. The image is representative of at least 10 different animals per experimental group. **(D)** Histological sections of *tibialis anterior* and diaphragm muscles stained with H & E. The images are representative of results obtained from at least five different animals per experimental group. Scale bar: 100 μm. Analyses were performed on animals at P120. qPCR analysis of mRNA levels for atrogin-1 and muRF1 in hind limb muscles at P10 **(E)** and *tibialis anterior* muscles at P30 **(F)**. Values are expressed as the fold change over wild-type. Each histogram represents the data obtained from at least five different animals per experimental group.

## Abbreviations

cGMP: cyclic guanosine monophosphate
CK: creatine kinase
CSA: cross sectional area
EBD: Evans blue dye
EDTA: ethylenediaminetetraacetic acid
EGTA: ethyleneglycoltetraacetic acid
FCCP: protonophore carbonylcyanide-p-trifluoromethoxyphenyl hydrazone
H & E: haematoxylin and eosin
L-NAME: L^ω^-arginine methylester
mTOR: mammalian target of rapamycin
mtDNA: mitochondrial DNA
Mul-1: mitochondrial E3 ubiquitin protein ligase 1
nNOS: NO synthases
NO: nitric oxide
OXPHOS: oxidative phosphorylation
P10/30/120: postnatal day 10/30/120
PKG: cGMP-dependent-protein kinases
qPCR: real-time quantitative PCR
SDS: sodium dodecyl sulphate
TEM: transmission electron microscopy
TMRM: tetramethylrhodamine, methyl ester
UPR^mt^: unfolded protein response
WBT: whole body tension
YFP: yellow fluorescent protein
