# Supplementary figures and images for: Deficient nitric oxide signalling impairs skeletal muscle growth and performance: involvement of mitochondrial dysregulation
Source: Skelet Muscle. 2014 Dec 12;4:22. doi: 10.1186/s13395-014-0022-6 (PMC4272808; doi:10.1186/s13395-014-0022-6)

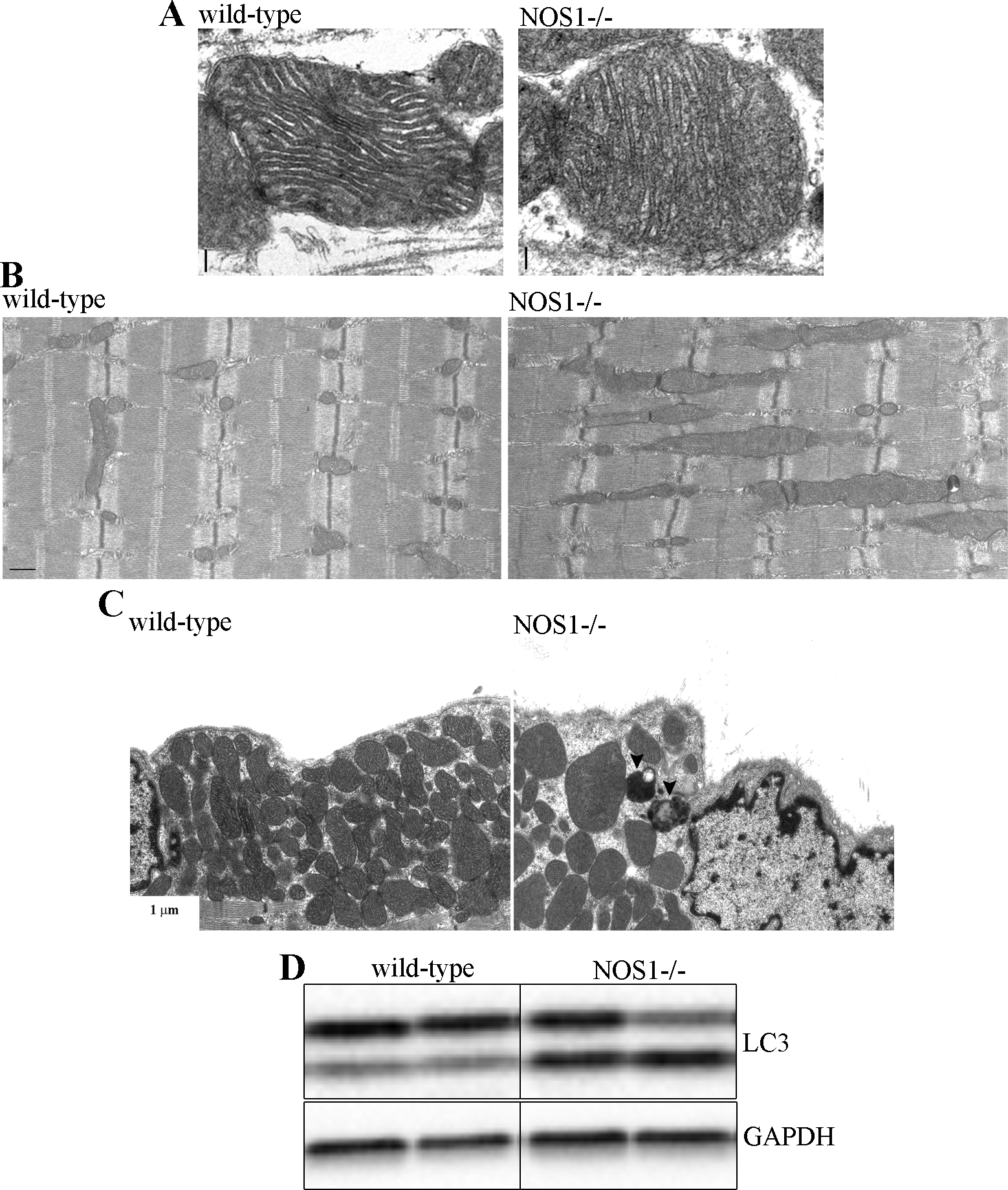

Supplement: Additional file 1: Figure S1. — Mitochondrial ultrastructure and LC3 lipidation in skeletal muscles of wild-type and NOS1-/- mice. (A) TEM images of subsarcolemmal mitochondria of tibialis anterior muscles. Scale bar: 0.1 μm. (B) TEM images of intermyofibrillar mitochondria of tibialis anterior muscles. Scale bar: 1 μm. (C) TEM images of diaphragm muscles detecting the presence of autophagic vacuoles (arrowheads) in NOS1-/- fibres. TEM images are representative of results obtained from at least three different animals per experimental group. (D) Western blot analysis of LC3 lipidation in diaphragm muscles of wild-type and NOS1-/- mice. GAPDH was used as internal standard. The image is representative of results obtained from at least 10 different animals per experimental group. Analyses were performed on animals at P120. [file 13395_2014_22_MOESM1_ESM.tiff]

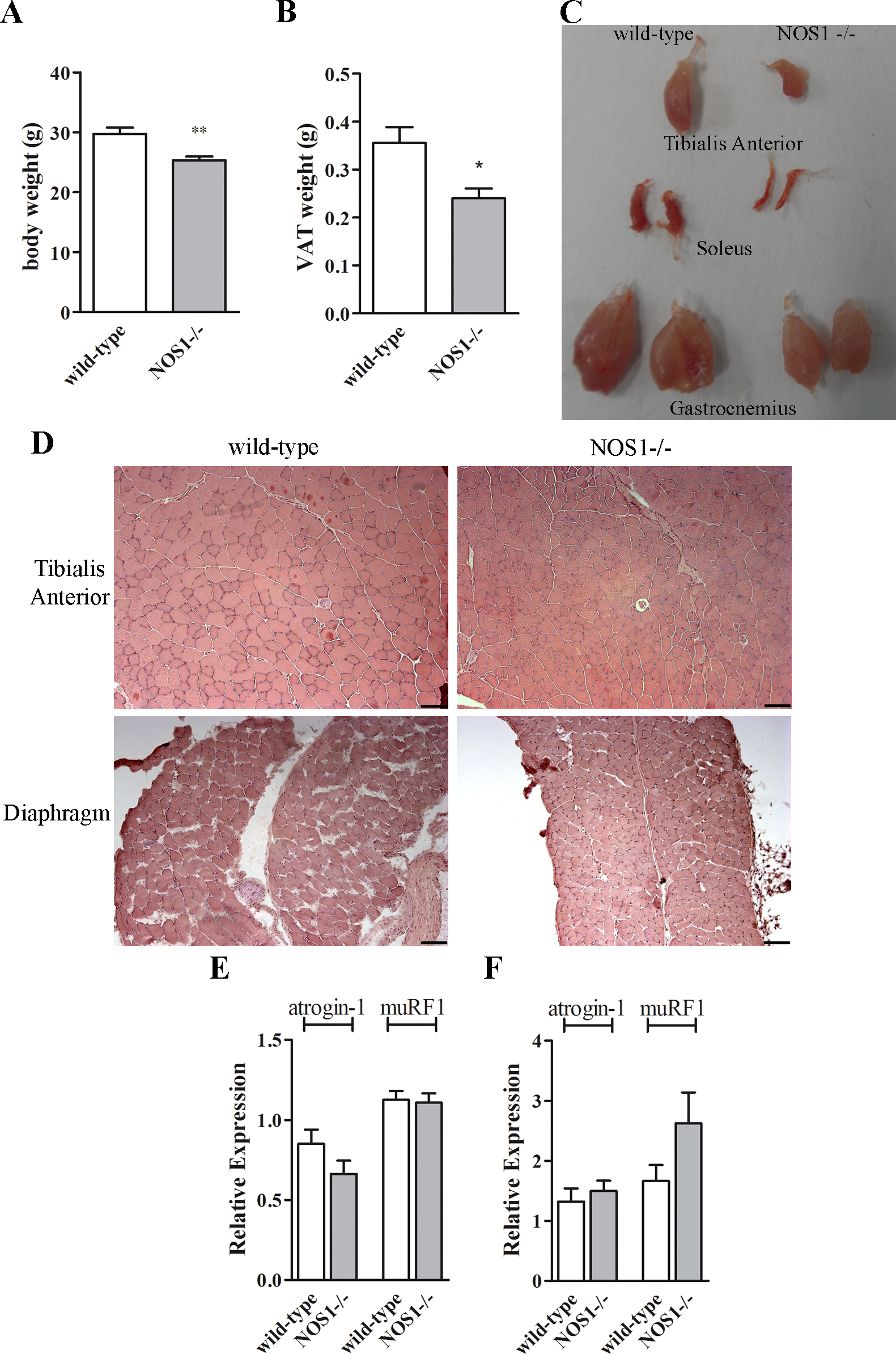

Supplement: Additional file 2: Figure S2. — Weight, muscle structure and muscle expression of ubiquitin ligases in wild-type and NOS1-/- mice. Body (A) and visceral adipose tissue (VAT) (B) weight. Each histogram represents the data obtained from at least three to eight different animals per experimental group. *P <0.05, and **P <0.01 versus the respective wild-type control. (C) Pictures of tibialis anterior, soleus, and gastrocnemius muscles. The image is representative of at least 10 different animals per experimental group. (D) Histological sections of tibialis anterior and diaphragm muscles stained with H & E. The images are representative of results obtained from at least five different animals per experimental group. Scale bar: 100 μm. Analyses were performed on animals at P120. qPCR analysis of mRNA levels for atrogin-1 and muRF1 in hind limb muscles at P10 (E) and tibialis anterior muscles at P30 (F). Values are expressed as the fold change over wild-type. Each histogram represents the data obtained from at least five different animals per experimental group. [file 13395_2014_22_MOESM2_ESM.tiff]
